# 4-Methylumbelliferone suppresses catabolic activation in anterior cruciate ligament-derived cells via a mechanism independent of hyaluronan inhibition

**DOI:** 10.1186/s13018-021-02637-6

**Published:** 2021-08-17

**Authors:** Masaru Idota, Shinya Ishizuka, Hideki Hiraiwa, Satoshi Yamashita, Hiroki Oba, Yusuke Kawamura, Takefumi Sakaguchi, Takahiro Haga, Takafumi Mizuno, Itaru Kawashima, Kanae Kuriyama, Shiro Imagama

**Affiliations:** Department of Orthopedic Surgery, Nagoya University Graduate School of Medicine, 65 Tsurumaicho Shouwaku Nagoya, Aichi, 4668550 Japan

**Keywords:** Anterior cruciate ligament, Osteoarthritis, 4-Methylumbelliferone, Hyaluronan, Matrix metalloproteinase, Interleukin-6

## Abstract

**Background:**

The anterior cruciate ligament (ACL) has a key role as a dynamic stabilizer of the knee joints, and ACL dysfunction caused by traumatic or degenerative rupture accelerates osteoarthritis progression. Thus, it is important to prevent the degenerative rupture of the ACL. 4-Methylumbelliferone (4-MU), a pre-approved drug, exerts anti-inflammatory effects in osteoarthritis chondrocytes. It was originally used as an inhibitor of hyaluronan synthesis in chondrocytes.

**Methods:**

In this study, we investigated whether 4-MU affects the expression of catabolic factors, such as matrix metalloproteinase (MMP)-1, MMP-3, and interleukin (IL)-6, in ACL-derived cells and ACL explant cultures using immunohistochemistry, real-time RT-qPCR, and capillary western immunoassay. Furthermore, the hyaluronan concentration was evaluated using a colorimetric assay. Statistical analyses were conducted using analysis of variance for multi-group comparisons, followed by Tukey or Tukey-Kramer post hoc test.

**Results:**

Our results revealed, for the first time, that 4-MU suppressed the IL-β-induced upregulation of pro-catabolic factors, such as MMP-1, MMP-3, and IL-6, in ACL-derived cells. This suppressive effect was also observed in the cultured ligament tissues in ex vivo experiments. 4-MU also reversed an enhanced dependence on glycolysis in IL-1β-activated ACL-derived cells. Furthermore, we found that the suppressive effects of 4-MU were exerted directly and not through the inhibition of hyaluronan synthesis.

**Conclusions:**

We conclude that 4-MU could be an effective and useful treatment for knee osteoarthritis, owing to its anti-inflammatory effect on, not only chondrocytes but also on ligament cells.

## Background

Osteoarthritis (OA) is the most common chronic disease in humans and is characterized by cartilage degeneration and osteophyte formation, which lead to pain and disability [1]. Previous studies have reported that various OA-related cytokines and enzymes induce inflammation and degeneration of all joint components, including the cartilage, synovia, menisci, and ligaments [2].

Among these intra-articular tissues, the anterior cruciate ligament (ACL) has a key role as a dynamic stabilizer of the knee joint, and previous studies have shown that ACL dysfunction caused by traumatic or degenerative rupture accelerates OA progression [1]. Usually, in young patients, ACL rupture occurs when the knee joint is subjected to excessive force during sporting activity. However, in elderly patients with knee OA, the inflammatory environment gradually causes ACL destruction due to degenerative histological changes [3]. Several studies reported that during total knee arthroplasty (TKA) in OA knee joints, 22 to 75% of the ACL is torn or deficient [4, 5]. Thus, severe degenerative changes following ACL rupture in the knee joint might enhance OA development synergistically [6].

Studies have reported various pathways and mediators that contribute to the cartilage degeneration and development of knee OA, such as proinflammatory cytokines (interleukin [IL]-1β, IL-6, and tumor necrosis factor [TNF]-α) and enzymes (matrix metalloproteinase [MMP]-1, MMP-3, and MMP-13 and a disintegrin and metalloproteinase with thrombospondin motifs [ADAMTS]-4 and ADAMTS-5) [7]. These OA-associated molecules play a crucial role in, not only cartilage degradation but also degenerative ligament rupture. However, the pathogenesis underlying the ligament degeneration is not fully understood.

It has been reported that most OA-associated cytokines and enzymes are synthesized and released from fibroblasts into the synovia [8]. These enzymes cause ligament degradation directly. They also induce additional cytokine synthesis, which affects the ligament cells during autocrine/paracrine signaling. As the ligaments are composed of various collagen fibers, MMPs, which are known to be collagenases, play a crucial role in degrading major components of the ligaments [2]. Thus, to prevent degenerative ligament rupture, suppression of MMP expression and its upstream mediators could be an important therapeutic approach.

In the last decade, various ACL preserved surgeries for knee OA have been increasing, such as high tibial osteotomy (HTO), uni-compartmental knee arthroplasty (UKA), and bi-cruciate retaining (BCR)-TKA. The indication for these surgeries is based on the pre-operative existence of a functional ACL. In addition, the ACL should be preserved postoperatively. There are some reports that ACL rupture caused severe complication including revision surgery after those ACL preserved surgeries. Therefore, for a good clinical outcome of ACL preserved surgeries for knee OA, it is necessary to prevent degenerative rupture of the ACL.

4-Methylumbelliferone (4-MU), a pre-approved drug, has been used as an inhibitor of hyaluronan (HA) biosynthesis in laboratory experiments, due to one of its abilities that sequesters intracellular UDP-glucuronic acid. Previously, we reported that 4-MU blocks the increase in the expression of MMP-13, ADAMTS-4, and other OA-related molecules in pro-catabolically activated human chondrocytes via an HA-independent mechanism [9].

Few studies have focused on inflammation in the ligament cells, and none have assessed whether 4-MU has an anti-inflammatory effect on ligament cells. The purpose of this study was thus to examine whether 4-MU affects the IL-β-enhanced expression of MMP-1, MMP-3, and IL-6 in ACL-derived cells.

## Methods

### Materials

Dulbecco’s modified Eagle’s medium (DMEM) (product no: D6046) and fetal bovine serum (FBS) (product no: F7524) were obtained from Sigma-Aldrich (St. Louis, MO, USA). IL-1β (catalog no: 201-LB) was purchased from R&D Systems, Inc. (Minneapolis, MN, USA). 4-MU (stock no: A10337) was procured from Alfa Aesar (Ward Hill, MA, USA). Pronase (catalog no: 537088; activity: ≥ 70,000 proteolytic units/g dry weight; Merck, Darmstadt, Germany) and collagenase P (catalog no: 11213865001; activity: > 1.5 U/mg lyophilizate; Roche, Mannheim, Germany) were used for dissociation of the tissues. Pharmaceutical grade high molecular mass HA (ARTZ) was purchased from Seikagaku Co. (Chiyoda-ku, Japan).

### Cell culture

Human ACL-derived cells were isolated from the ACL obtained during TKA of patients with knee OA at Nagoya University Hospital. The patients, 55% female and 45% male, had an average age of 72.1 ± 8.7 years. Human ACL-derived cells were liberated from the ACL by sequential pronase/collagenase P digestion. Cells were plated as monolayers (0.25–0.30 × 10^5^ cells/cm^2^) and cultured in DMEM containing 10% FBS, 100 μg/mL streptomycin, 100 units/mL penicillin, and 0.25 μg/mL amphotericin. Confluent cultures of cells were incubated overnight with medium containing 1% FBS; then, they were incubated in serum-free medium for 1 h, following which they were treated with 0.1 ng/mL IL-1β in fresh serum-free culture medium with or without 1.0 mM of 4-MU for 12 h.

### ACL explant culture

Human ACLs were harvested during the TKA of patients with knee OA at Nagoya University Hospital. Harvested ACLs were cultured without or with 2.0 ng/mL IL-1β in the presence or absence of 1.0 mM 4-MU for 1 week. For analysis, the treated explants were fixed with 4% buffered paraformaldehyde overnight at 4 °C, rinsed in 30% sucrose phosphate-buffered saline, and embedded in paraffin.

### Cytotoxicity of LDH assay

Cytotoxicity of 4-MU was evaluated colorimetrically by the activity of the lactate dehydrogenase (LDH) released in the media, using cytotoxicity LDH Assay Kit (Dojindo, Kumamoto, Japan). Briefly, cells were treated with different concentrations of 4-MU (0 to 16 mM) for 12 h, the liquid supernatant was collected and LDH activity was detected according to the manufacturer’s instruction.

### Immunohistochemistry

Paraffin-embedded ACLs were sectioned longitudinally (5 μm thick). In order to perform the staining procedure, first these sections were deparaffinized, rehydrated, and then antigen retrieval was accomplished by 10 mM citrate buffer at 60 °C for 1 h. These sections were incubated with rabbit primary antibodies against MMP-1 (1:100), MMP-3 (1:50), or IL-6 (1:400) at 4 °C overnight, rinsed with phosphate-buffered saline, and incubated with reagents included in the Histofine SAB-PO(R) kit (Nichirei Biosciences Inc., Tokyo, Japan). All sections were visualized using the Olympus BX53 microscope (Olympus, Tokyo, Japan); images were captured digitally using the Leica DFC7000 T digital camera and processed using the Leica Application Suite Ver4.6 imaging software. The primary antibodies used were rabbit anti-MMP1 antibody (1:100, ab52631, lot: GR3261996-3; Abcam, Cambridge, UK), rabbit anti-MMP3 antibody (1:50, ab52915, lot: GR3249690-2; Abcam), and rabbit anti-IL-6 antibody (1:400, ab6672, lot: GR3195128-25; Abcam). The acquired images were analyzed using ImageJ Fiji (https://imagej.net/software/fiji/). Color deconvolution in ImageJ Fiji was used to separate the 3,3′-diaminobenzidine staining, and the mean gray values in the nucleus and cytoplasm were measured and quantified.

### Real-time qRT-PCR

Total RNA was extracted using the RNeasy Mini Kit (catalog no: 74106, Qiagen, Hilden, Germany). After reverse transcription, real-time qRT-PCR was performed for evaluation target gene expression. Primer sequences for *MMP-1*, *MMP-3*, *IL-6*, and *glyceraldehyde 3-phosphate dehydrogenase* (*GAPDH*) (Sigma-Aldrich) are listed in Table 1. The High Capacity cDNA Reverse Transcription Kit was obtained from Applied Biosystems (Waltham, MA, USA), and LightCycler®480 SYBR® Green reagents were procured from Roche. The fold increase in RNA copy numbers was calculated as a relative ratio of the expression of the target gene to that of *GAPDH* (ΔΔCt method).

**Table 1:**
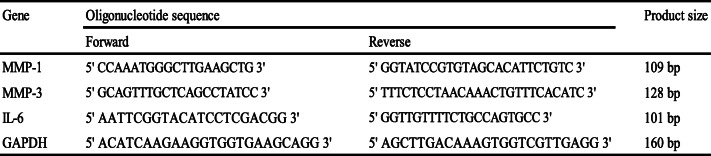
List of primer sequences used in the study

*MMP*, matrix metalloproteinase; *IL*, interleukin; *GADPH*, glyceraldehyde 3-phosphate dehydrogenase

### Capillary western immunoassay (JESS)

Protein expression was evaluated by capillary western immunoassay (JESS) using cell lysates. Total protein was extracted using Cell Lysis Buffer (Cell Signaling Technology, Danvers, MA, USA) containing a protease inhibitor cocktail. The immunoassay was performed on the JESS system (004–650, Protein Simple, a Bio-Techne Brand, San Jose, CA, USA) according to the manufacturer’s instructions using a 12–230 kDa separation module (SM-W004, Protein Simple, a Bio-Techne Brand) and an anti-rabbit detection module (DM-001, Protein Simple, a Bio-Techne Brand). The plate of the separation module was loaded and placed in the device. The reactions were run in the capillary system, the proteins were identified by specific antibodies, and their chemiluminescence reactions were measured and the digital blot images were captured. Specific antibodies used for the capillary western immunoassay were rabbit anti-MMP1 (1:50, catalog no: 54376, lot1; Cell Signaling Technology), rabbit anti-MMP3 (1:100, catalog no: 14351, lot1; Cell Signaling Technology), rabbit anti-IL-6 (1:50, catalog no: 12153, lot3; Cell Signaling Technology), and rabbit anti-β-actin (1:100, catalog no: 4970, lot15; Cell Signaling Technology).

### Colorimetric assay

Cells were incubated for 12 h with 0.1 ng/ml IL-1β in fresh serum-free culture medium with or without 1.0 mM 4-MU. The culture supernatant fluids were collected and the HA was quantified by enzyme-linked sandwich assay (Hyaluronan DuoSet ELISA, DY3614-05, R&D Systems) following the manufacturer’s instructions. The lactate concentration in culture supernatants was determined using l-Lactate Assay Kit I (Eton Bioscience, Research Triangle, NC, USA) according to the manufacturer’s instructions.

### Metabolomic analyses using a flux analyzer

ACL-derived cells were plated at 1.5 × 10^4^ cells/well into specially designed Seahorse XFp cell culture miniplates (Agilent Technologies, Santa Clara, CA, USA). The confluent monolayers were incubated for 12 h without or with 0.1 ng/mL IL-1β in the presence or absence of 1.0 mM 4-MU. Prior to the assay, the medium was changed to serum-free Seahorse XF DMEM (without phenol red but with 10 mM glucose, 1.0 mM pyruvate, and 2.0 mM glutamine) of pH 7.4. The cells were then mated with sensor cartridges and analyzed on the Seahorse XFp flux analyzer (Agilent Technologies) using the Seahorse XFp Real-Time ATP Rate Assay Kit (103591-100) according to the manufacturer’s guidelines.

### Phosphorylation of NF-κB and AKT

The NF-κB and AKT phosphorylation activity in interleukin (IL)-1β treatment of anterior cruciate ligament (ACL)-derived cells were measured. In brief, cells were treated with IL-1β in the absence or presence of 1 mM 4-MU, and cell lysate were collected and used for Western blotting analysis with the antibodies of rabbit anti-AKT (1:20, catalog no: 9272, lot22; Cell Signaling Technology), rabbit anti-phospho-AKT (1:20, catalog no: 9271, lot12; Cell Signaling Technology), rabbit anti-NF-κB (1:60, catalog no: 8242, lot16; Cell Signaling Technology), and rabbit anti- phospho-NF-κB (1:60, catalog no: 3033, lot17; Cell Signaling Technology).

### Statistical analysis

Data are presented as mean ± standard deviation. All statistical analyses were performed with EZR (Saitama Medical Center, Jichi Medical University, Saitama, Japan), which is a graphical user interface for R (The R Foundation for Statistical Computing, Vienna, Austria). More precisely, it is a modified version of R commander designed to add statistical functions frequently used in biostatistics. All data were obtained from at least three independent experiments performed in duplicate or triplicate. For multiple group comparisons, analysis of variance (ANOVA) was performed, followed by Tukey or Tukey-Kramer post hoc tests. A *p* value < 0.05 was considered to denote statistical significance.

## Results

### Induction of inflammation

**Fig. 1:**
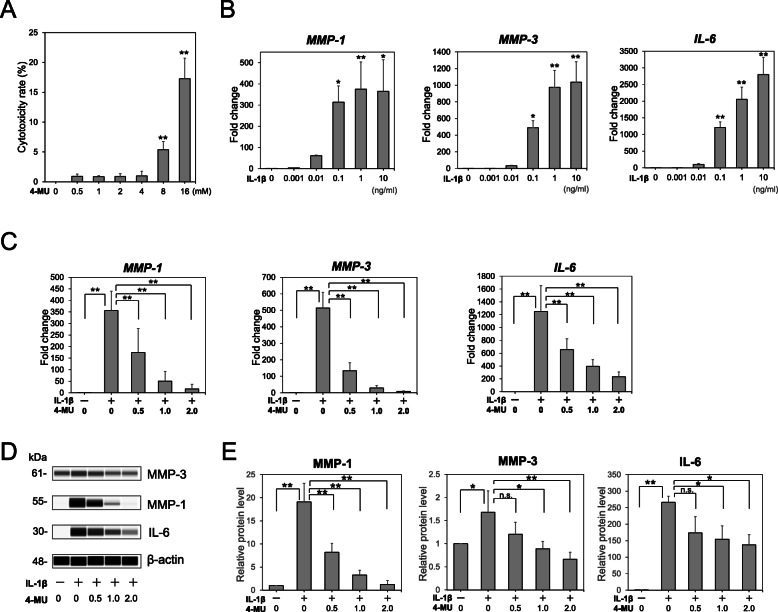
Effect of 4-methylumbelliferone (4-MU) on the anterior cruciate ligament (ACL)-derived cells stimulated with interleukin (IL)-1β. ACL-derived cells were treated with 0 to 10 ng/mL of IL-1β for 12 h. (**A**) To examine cytotoxicity of 4-MU, ACL-derived cells were treated with different concentrations of 4-MU for 12 h and cytotoxicity LDH Assay was performed (*n* = 3). (**B**) qRT-PCR was performed to determine the mRNA expression of matrix metalloproteinase 1 (*MMP-1*), *MMP-3*, and *IL-6* (*n* = 3). ACL-derived cells were treated with or without 0.1 ng/mL IL-1β in the presence or absence of different concentrations of 4-MU for 12 h. (**C**) qRT-PCR (*n* = 6) and (**D**) capillary western immunoassay (*n* = 3) were performed to determine the *MMP-1*, *MMP-3*, and *IL-6* mRNA and MMP-1, MMP-3, and IL-6 protein expression, respectively. (**E**) Statistical quantification of each protein expression. The values are presented as mean ± SD. *p < 0.05; **p < 0.01. n.s., not significant

To decide the optimal concentration for inducing an inflammatory response, ACL-derived cells were treated with different concentrations of IL-1β. The levels of mRNA expression of MMP-1, MMP-3, and IL-6 in human ACL-derived cells trends increase after a 12-h treatment with IL-1β (0.001–10 ng/mL) (Fig. 1A).

### Cytotoxicity of 4-MU to ACL-derived cells

To examine cytotoxicity of 4-MU to ACL-derived cells, we measured LDH release from cultured cells. Significantly higher cytotoxicity of 4-MU was observed more than 8 mM concentration of 4-MU treatment for 12 h (Fig. 1A). Thus, less than 4 mM concentration of 4-MU seems to be non-cytotoxic.

### Suppressive effect of 4-MU on MMP-1, MMP-3, and IL-6 expression in the IL-1β-stimulated ACL-derived cells

Based on the induction of the inflammatory response, the human ACL-derived cells were treated with IL-1β and showed a pronounced stimulation of *MMP-1*, *MMP-3*, and *IL-6* mRNA expression (Fig. 1B). To examine the effect of 4-MU on the expression of these genes, cells were co-incubated with IL-1β and 4-MU. When cells were co-incubated with IL-1β and 4-MU, the mRNA and protein expression of MMP-1, MMP-3, and IL-6 were decreased (Fig. 1B‑D). We found that 1.0 mM of 4-MU was sufficient to inhibit the expression of MMP-1, MMP-3, and IL-6; thus, this concentration was used for the following experiments.

### Immunohistochemical findings

**Fig. 2:**
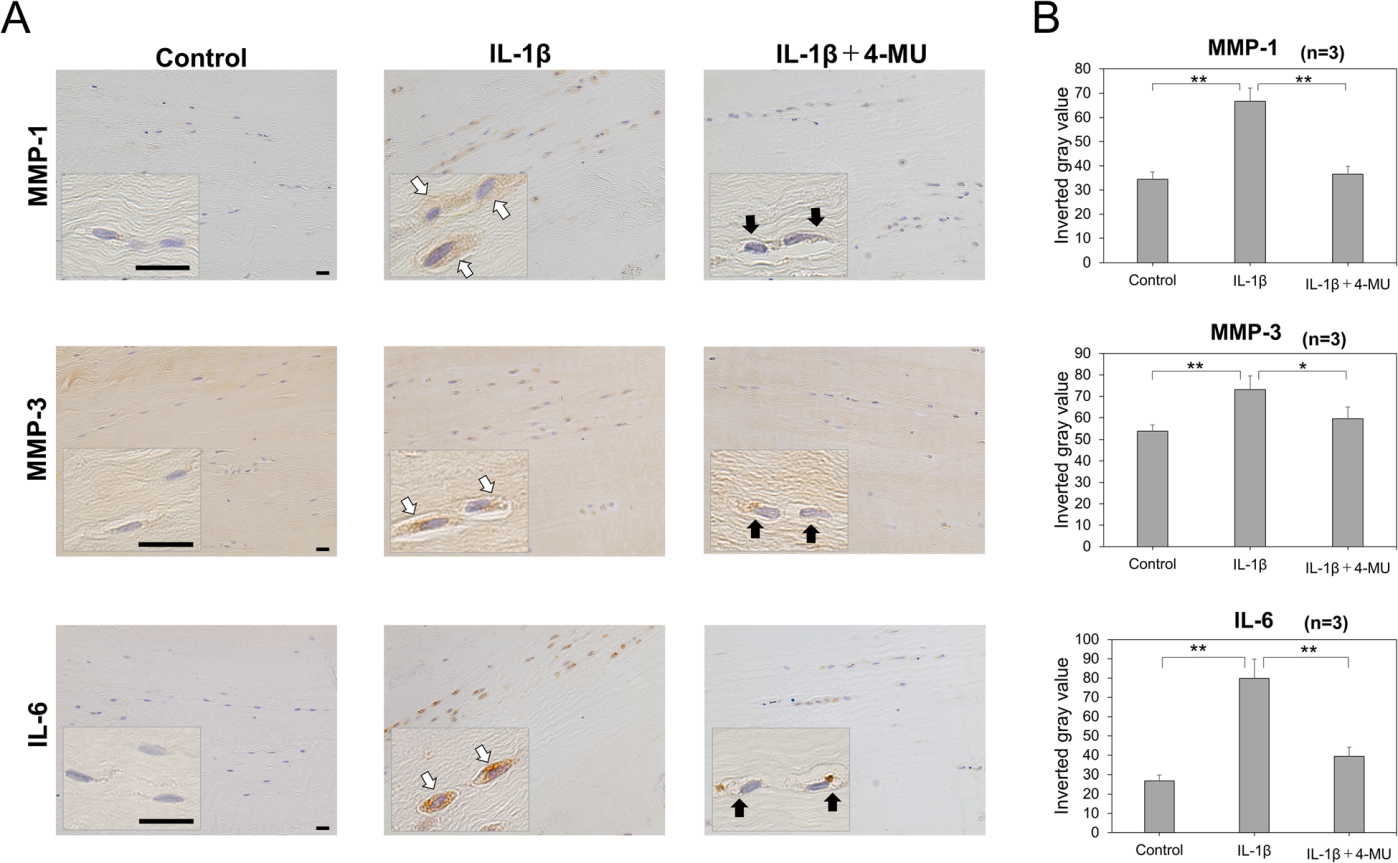
Matrix metalloproteinase (MMP)-1, MMP-3, and interleukin (IL)-6 immunohistochemistry in the anterior cruciate ligament (ACL) explants. ACL explants were cultured in a medium with or without 2.0 ng/mL IL-1β in the presence or absence of 1.0 mM 4-MU for 7 days. Here, we show the representative images of MMP-1, MMP-3, and IL-6 staining of the ACL explants. Scale bars represent 20 μm. Staining for MMP-1, MMP-3, and IL-6 indicated the highest protein expression in the 2.0 ng/mL IL-1β-treated explants (white arrows). The expression in explants that were co-treated with 2.0 ng/mL IL-1β and 1.0 mM 4-MU was substantially reduced (black arrows). (**B**) Statistical quantification for staining positivity of each antibody (*n* = 3). The values are presented as mean ± SD. *p < 0.05; **p < 0.01

To determine the biological effects of 4-MU on the ACL tissue, the human ACL explants were cultured with or without IL-1β in the presence or absence of 4-MU. After 7 days, immunohistochemistry for MMP-1, MMP-3, and IL-6 was performed. Staining for MMP-1, MMP-3, and IL-6 exhibited the highest protein expression in 2.0 ng/mL IL-1β-treated explants. The expression in explants that were co-treated with 2.0 ng/mL IL-1β and 1.0 mM 4-MU was significantly reduced (Fig. 2).

### Effect of 4-MU-mediated inhibition of HA synthesis

**Fig. 3:**
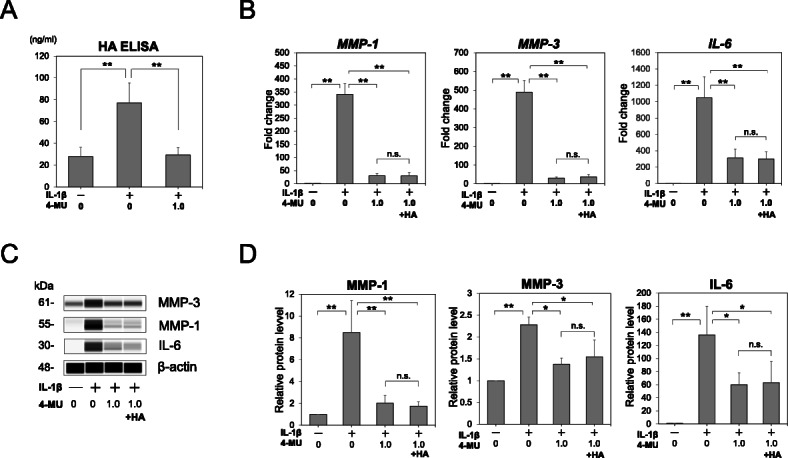
The effect of exogenous hyaluronan (HA) on inhibition of interleukin (IL)-1β by 4-methylumbelliferone (4-MU). (**A**) The HA concentrations in media (ng/mL) were determined using the HA ELISA (*n* = 6). Exogenous HA (1.0 mg/mL) was added to the cultured anterior cruciate ligament (ACL)-derived cells during incubation without or with 0.1 ng/mL IL-1β in the presence or absence of 1.0 mM 4-MU. After a 12 h incubation, the cells were lysed and analyzed by (**B**) real-time qRT-PCR (*n* = 6) and (**C**) capillary western immunoassay for determining the *MMP-1*, *MMP-3*, and *IL-6* mRNA and matrix metalloproteinase (MMP)-1, MMP-3, and IL-6 protein expression, respectively. (**D**) Statistical quantification of each protein expression (*n* = 3). The values are presented as mean ± SD. *p < 0.05; **p < 0.01. n.s., not significant

The ability of the ligament-derived cells to synthesize HA was evaluated using ELISA. HA production was also observed in ACL-derived cells, and induction of inflammation by IL-1β enhanced HA synthesis. Furthermore, HA synthesis in cells treated with IL-1β was suppressed by 4-MU treatment (Fig. 3A).

### 4-MU directs inhibition of pro-catabolic events in an HA-independent manner

To examine the requirement of HA alterations for the suppression of MMP-1, MMP-3, and IL-6 expression by 4-MU, we investigated the mechanistic approaches for independently modifying HA. As shown in Fig. 3B and C, exposure of ACL-derived cells to IL-1β resulted in the stimulation of MMP-1, MMP-3, and IL-6 expression in cell layers. However, this enhanced production of MMP-1, MMP-3, and IL-6 was blocked by 4-MU. Exogenous pharmaceutical grade HA (1.0 mg/mL) was added to the culture media to compensate for the reduced HA production by 4-MU. However, no rescue of *MMP-1*, *MMP-3*, and *IL-6* mRNA and MMP-1, MMP-3, and IL-6 protein production was observed (Fig. 3B‑D).

### 4-MU reverses an enhanced dependence on glycolysis in IL-1β-activated ACL-derived cells

**Fig. 4:**
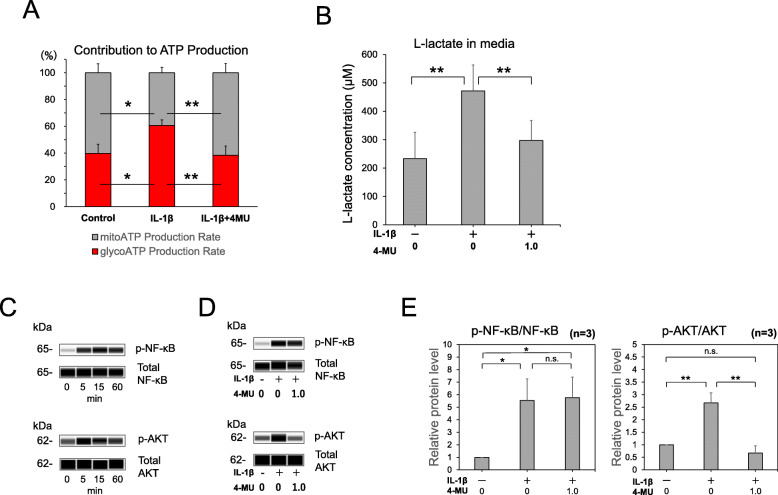
4-Methylumbelliferone (4-MU) reverses enhanced glycolysis dependence in the interleukin (IL)-1β-activated anterior cruciate ligament (ACL)-derived cells. ACL-derived cells were incubated for 12 h with or without IL-1β (0.1 ng/mL) in the presence or absence of 4-MU (1.0 mM). (**A**) The data from the ATP rate assay, which evaluates the contribution rates of the glycolysis (red bars; *n* = 3) and mitochondrial respiration (gray bars; *n* = 3) toward ATP production, are shown as stacked bars. (**B**) The concentration of l-lactate in the conditioned medium from the culture of the ACL-derived cells treated with IL-1β and 4-MU is indicated. To examine the 4-MU effect on early signaling events during interleukin (IL)-1β treatment of anterior cruciate ligament (ACL)-derived cells. (**C**) Phosphorylation of nuclear factor-kappa B (NF-κB) and AKT at 0-60 min in IL-1β treated ACL-derived cells. (**D**) Phosphorylation of NF-κB at 15 min and AKT at 5min in IL-1β treated ACL-derived cells in the absence or presence of 1 mM 4-MU. (**E**) For quantification, phospho NF-κB and AKT was normalized to total-NF-κB or AKT, respectively and presented as values relative to control (*n* = 3). The values are presented as mean ± SD. *p < 0.05; **p < 0.01

In ACL-derived cells, the inhibition of HA biosynthesis (via 4-MU) inhibits the expression of MMPs and IL-6. To determine the potential characteristics of the 4-MU inhibitory mechanisms, the energy metabolism in ACL-derived cells was examined. As shown in Fig. 4A, the Seahorse ATP rate assay enabled simultaneous visualization of the contributions of glycolysis and mitochondrial respiration in ACL-derived cell ATP production. In the control ACL-derived cells, glycolysis contributed to less than half of the ATP production. IL-1β-activated ACL-derived cells showed a reciprocal phenomenon of enhanced glycolysis and decreased contribution of mitochondria to ATP production. These changes were reversed by treatment with 4-MU (Fig. 4A). As shown in Fig. 4B, a lactate assay was performed to measure l-lactate in the culture medium. IL-1β treatment enhanced the lactic acid release into the culture medium compared with that in the control culture. And more important, the IL-1β-enhanced lactic acid release were significantly suppressed by 4-MU (Fig. 4B).

### Phosphorylation of NF-κB and AKT

To examine the 4-MU effect on phosphorylation of NF-κB and AKT, early signaling events during interleukin (IL)-1β treatment of anterior cruciate ligament (ACL)-derived cells, the peak time-point of NF-kB and PI3K/AKT phosphorylation in cells treated with IL-1 β. IL-1β treatment resulted in prominent phosphorylation of NF-kB at 15 min, and AKT at 5 min, respectively(Fig. 4C). Since both phosphorylation became reduced after the time-point, we examined the 4-MU effect on phosphorylation of NF-kB at 15 min and PI3K/AKT at 5 min. As a result, phosphorylation of AKT, not NF-kB was suppressed by 4-MU treatment, and the suppression was statistically significant.

## Discussion

Here, we demonstrated for the first time that the IL-1β-induced expression of MMP-1, MMP-3, and IL-6 was suppressed by 4-MU treatment in a dose-dependent manner in human ACL-derived cells. Furthermore, the suppressive effect of 4-MU on pro-catabolic factors was confirmed in the tissue culture model. The inhibitory effect of 4-MU could be independent of a decrease in the pericellular HA.

Although the precise mechanism underlying ligament rupture in knee OA remains unclear, some studies suggest that intra-articular inflammation and the following degeneration of the ligament could be associated with ligament rupture. ACL preservation surgeries for knee OA, such as HTO, UKA, and BCR-TKA, are being performed increasingly. Although a few clinical studies have shown good results after UKA for ACL-deficient knees [10, 11], most studies showed high failure rates after UKA or HTO for ACL-deficient knees. Thus, clinical trials of concomitant or postoperative ACL reconstruction with HTO or UKA have been considered [12, 13]. Therefore, the fundamental prerequisite for a better clinical outcome in these surgeries is that the ACL is functionally intact. Considering the current transition of surgical treatment for knee OA to ACL preservation surgeries, inflammation in the ligament and subsequent degenerative rupture could be a therapeutic target in the following decade.

Various mediators and pathways that may directly affect intra-articular inflammation have been reported previously, including proinflammatory cytokines and enzymes that induce local tissue damage [2, 8]. The MMPs are expressed by synoviocytes, chondrocytes, and meniscus cells in knee OA. MMPs are composed of zinc-dependent endopeptidases with a particular specificity for degradation of the extracellular matrix (ECM) components of various intra-articular tissues [14]. The MMPs play a crucial role in ECM re-modeling by degrading collagenous ECM proteins, including various collagen proteins in the cartilage and ligaments. The fibrous tissue of the ligament is composed of various collagen fibers, including collagen types I, II, and III–VI [15], which can be degraded by MMPs [16].

In this study, we focused on MMP-1 and MMP-3 expression. Among various knee OA-related MMPs, collagenase (MMP-1) and stromelysin (MMP-3) are two of the most extensively studied enzymes [17]. Collagen network in the ligaments is predominantly composed of type I collagen [15]. MMP-1 is an interstitial collagenase that can degrade interstitial collagen, including type I collagen. Moreover, previous studies have reported a positive correlation between MMP-1 and cell apoptosis in experimental knee OA [18]. MMP-3 degrades collagen in various tissues in the knee joint and activates pro-MMP-1 [19]. It has been demonstrated that the levels of MMP-1 and MMP-3 are elevated in the synovial fluid of OA knees [20], and high protein levels of these MMPs are mostly reported to accelerate OA pathogenesis due to cartilage degradation [21, 22].

Presumably, MMPs released into the synovial fluid in OA knees diffuse, not only to the cartilage but also to the adjacent tissues, such as the meniscus and ligaments to cause further degradation. Some studies have reported that an association between elevated MMP levels and ECM degradation of the menisci [23]. Other studies have demonstrated increased MMP expression in the ligament cells induced by proinflammatory cytokines in the periodontal ligament cells [24]. However, few studies have reported the inflammatory effects of cytokines and enzymes on the knee ligament cells. Recently, Wang et al. [25] simulated an injured ligament model and showed that MMP-1, MMP-2, and MMP-3 expression are upregulated by IL-1β treatment in ACL-derived cells. In the present study, we showed the suppressive effect of a specific drug, 4-MU, on the increased MMP expression induced by IL-1β in ACL-derived cells obtained from OA knee joints.

We found that IL-6 expression was enhanced by IL-1β treatment in ACL-derived cells. IL-6 is among the main proinflammatory cytokines responsible for the cartilage degradation. IL-6 levels have been reported as being elevated in the synovial fluid of ACL-injured knee [26] and OA knee [27] and to induce various pro-catabolic factors in chondrocytes [28]. Alonso et al. [29] reported that the synovial fluid levels of IL-6 are significantly higher in OA knee tissues compared with those in control knee tissues. Makki et al. [30] reported that IL-6 inhibition suppresses IL-1β-induced MMP-3 and MMP-13 expression in human OA chondrocytes. Our results suggested that ACL degradation might be caused, at least partially, by autocrine IL-6 expression in ligament cells, and 4-MU could block the expression of inflammatory mediators in ligament cells.

4-MU has already been approved with a benign safety profile [31]. It has been used in many studies as an inhibitor of HA biosynthesis [32]. The mechanistic pathway for inhibiting HA synthesis by 4-MU involves the formation of 4-MU-glucuronic acid (GlcUA) glycoconjugates, which results in the depletion of cellular UDP-GlcUA. Previously, we have reported the anti-inflammatory effects of 4-MU in chondrocytes, which are mediated independently of the inhibition of HA synthesis [9]. Our present findings demonstrated that HA was produced in ACL-derived cells, and its synthesis increased by IL-1β treatment. Increase in HA production by IL-1β treatment in chondrocytes has been reported in previous studies [33]. However, few studies have confirmed HA expression in tenocytes or ligament cells [34]. To determine whether depletion of HA was required for 4-MU-mediated inhibition of MMPs and IL-6 expression, we explored mechanistic approaches to independently modify HA. Our quantitative analysis showed that 4-MU inhibited HA biosynthesis in ACL-derived cells. To compensate the HA loss by 4-MU, 1.0 mg/ml exogenous HA was added; however, the HA addition did not reverse the suppressive effect of 4-MU. These results demonstrate that the inhibitory effects of 4-MU on the inflammatory response in ACL-derived cells are mediated directly, and not via suppression of HA synthesis.

It has been recently suggested that metabolic changes in cells occur in some chronic diseases, including OA. Recent studies have reported that OA pathogenesis involves a metabolic switch toward glycolysis [35], and the upregulated glycolysis in OA chondrocytes can be a new therapeutic target for inflammatory diseases. Hence, to understand the mechanism underlying the effects of 4-MU, we focused on cellular metabolism and confirmed that 4-MU effectively reduced an enhanced dependence on glycolysis in the IL-1β-activated ACL-derived cells. We also evaluated l-lactate, a metabolite in glycolysis, in the culture medium and confirmed that its production was enhanced by IL-1β and suppressed by 4-MU. Our previous study have reported that OA model mice fed 4-MU exhibited less OA progression and reduced MMP-3 and MMP-13 immunostaining. 4-MU blocked IL-1β induced lactate production in cartilage explants [36]. Although there might be no consensus about in vivo OA model, the results from present study in ACL-derived cells are in accordance with our previous results in vivo mice experiments.

Our results demonstrated that the suppression of glycolysis secondarily enhanced mitochondrial respiration in ACL-derived cells as well as chondrocytes. It is hypothesized that such a cellular metabolic change, such as glycolysis reduction, is due to the depletion of the cellular UDP-GlcUA by 4-MU. In addition, our results showed AKT phosphorylation was suppressed by 4-MU. Previous studies reported the inhibition of AKT activation by 4-MU in cancer cell line, such as prostate cancer [37], osteosarcoma [38], and breast cancer [39]. Recent study reported that MMP-3 and -13 was induced by IL-1β via PI3K/AKT signaling pathways in chondrocytes [40]. In tumor cells, PI3K/Akt signaling pathway is reported to involve cell proliferation and apoptosis via CD44/HA interaction [41]. However, our results showed the suppressive effect of 4-MU on MMPs in ligament-derived cells is HA-independent. Thus, in primary ligament derived cells, not tumor cells, AKT phosphorylation could be inhibited by 4-MU independent of HA. However, further studies are necessary to elucidate the exact mechanism underlying this phenomenon.

Nevertheless, this study has some limitations. First, the ACL-derived cells could not be confirmed to be a homogenous population. This cell population might include both synoviocytes and ligament cells. However, most previous studies using ACL-derived cells have isolated the cells using a method similar to the one used here [15, 42]. Second, the ACL-derived cells and ACL tissues were obtained from OA knees to examine the 4-MU effect in the OA environment. Hence, the observed 4-MU effect might be limited to ACL-derived cells in OA. Third, the biological variation among the donors might be considerable.

## Conclusions

Our study showed that 4-MU exerts a suppressive effect on IL-1β-induced MMP-1, MMP-3, and IL-6 expression in ACL-derived cells. We conclude that 4-MU could be an effective treatment for knee OA, due to its anti-inflammatory effect on, not only chondrocytes but also on knee ligaments.

## Data Availability

All data generated or analyzed during this study are included in this published article.

## Abbreviations

OA: Osteoarthritis
ACL: Anterior cruciate ligament
IL: Interleukin
TNF: Tumor necrosis factor
MMP: Matrix metalloproteinase
ADAMTS: A disintegrin and metalloproteinase with thrombospondin motifs
HTO: High tibial osteotomy
UKA: Uni-compartmental knee arthroplasty
BCR: Bi-cruciate retaining
4-MU: 4-Methylumbelliferone
HA: Hyaluronan
TKA: Total knee arthroplasty
DMEM: Dulbecco’s Modified Eagle Medium
FBS: Fetal bovine serum
ECM: Extracellular matrix
